# A Data-Driven Threshold Standard for Olfactory Behavior: A Case Study with *Trichogramma japonicum*

**DOI:** 10.3390/insects17080798

**Published:** 2026-07-31

**Authors:** An-Na Yang, Yu-Xin Feng, Ke Li, Hang Li, Su Wang, Yuan-Xi Li

**Affiliations:** 1 Key Laboratory of Integrated Management of Crop Disease and Pests, Ministry of Education, Department of Entomology, College of Plant Protection, Nanjing Agricultural University, Nanjing 211800, China; 13488867972@163.com (A.-N.Y.); 2023802191@stu.njau.edu.cn (Y.-X.F.); 2023202053@stu.njau.edu.cn (K.L.); 2024802139@stu.njau.edu.cn (H.L.); 2 State Key Laboratory of Agricultural and Forestry Biosecurity, Nanjing Agricultural University, Nanjing 211800, China; 3 Institute of Plant Protection, Beijing Academy of Agriculture and Forestry Sciences, Beijing 100097, China; wangsu@baafs.net.cn

**Keywords:** behavior threshold, four-arm olfactometer, olfactory preference, oviposition choice, *Trichogramma*

## Abstract

Objectively defining an effective choice is crucial for the reliability of results in insect olfactory behavior research. Relying on subjective time thresholds often leads to inconsistent findings, primarily due to the high likelihood of mistaking an accidental intrusion for an effective choice. Our analysis indicated that 29% of preference responses were not consistent among 0 s, 15 s, 30 s, and 60 s, when these thresholds were applied to evaluate the behavioral responses of *Trichogramma japonicum* Ashmead (Hym.: Trichogrammatidae) to 20 odorants in a four-arm olfactometer. This inconsistency arises because subjective thresholds fail to distinguish between accidental, transient entries and sustained, purposeful choices, leading to false positives or false negatives. To address this issue, we compiled a database of the initial stay duration of *T. japonicum* in the odor zone, and applied the 5th percentile of its empirical distribution—a widely accepted statistical standard for excluding extreme outliers (i.e., accidental brief entries) in medical and biological research—as the threshold to differentiate between accidental intrusion and effective choice. We then systematically assessed the behavioral responses of *T. japonicum* to 20 odorants and validated the authenticity of olfactory preferences through oviposition choice experiments. A continuous stay of ≥10 s was determined as an effective choice. Based on this criterion, *T. japonicum* exhibited significant preference for 0.1 μg/μL α-pinene, 0.01 μg/μL D-limonene and 0.001 μg/μL citral, whereas avoiding 10 μg/μL β-ionone. The results of the oviposition choice experiments showed high consistency with the olfactory behavioral preferences, confirming the reliability of this standard in predicting its actual host selection decisions. Collectively, this study proposes a data-driven protocol for establishing an objective and stable behavioral criterion in insect olfactory behavior research.

## Simple Summary

*Trichogramma japonicum* is a tiny parasitic wasp widely used to control rice pests. In laboratory studies, researchers often place these wasps in a four-arm olfactometer to test which odors attract them. However, different researchers use different rules to decide whether a wasp’s visit to an odor is a real “choice” or just a random accidental entry—some count any visit, while others require the wasp to stay for 15, 20, 30, or even 60 s. This lack of a unified standard makes it difficult to compare results across studies. In this study, we analyzed the behavior of 3000 female wasps and found that applying different rules can change the experimental conclusions in 29% of cases. Based on the distribution of the wasps’ actual stay durations, we established a data-driven standard: a continuous stay of 10 s or more should be considered a genuine choice. We then validated this standard through egg-laying preference experiments, confirming that it reliably predicts the wasps’ real host-selection behavior. This study provides an objective, reproducible method for defining insect behavioral responses, which will help standardize future research in chemical ecology and biological control.

## 1. Introduction

Research on insect olfactory behavior is crucial for understanding chemical communication, host location, and ecological adaptation [1,2]. In studies investigating insect behavioral preferences towards odorants, the Y-tube olfactometer, as one of the earliest technical tools [3], has been widely used [4,5,6,7,8]. Later, a four-arm olfactometer that creates clearly distinct, contiguous odor fields that can be easily entered, left and re-entered by walking insects was designed [9]. Furthermore, it supports the simultaneous presentation of multiple odors or different concentrations of an odor, enabling the study of complex olfactory preference scenarios. Consequently, it is widely regarded as a means to more realistically simulate natural conditions [9,10]. Both Y-tube and four-arm olfactometers have been used to investigate olfactory response behavior of various insects including hymenopterous parasitoids and small insects since they were designed [11,12,13,14,15,16,17,18,19,20], and a search on Web of Science yields a vast number of publications utilizing the four-arm olfactometer.

When using a four-arm olfactometer, the underlying hypothesis is that the insect will move towards and remain in arms emitting preferred odors. A specific area or length was set to indicate that a behavioral response occurred when the observed insect entered the area or crossed the length [9,10]. First choice and residence time were mostly used in studies assessing insect behavioral preference [21,22,23,24,25,26,27,28,29,30,31]. Ideally, an insect would move from a random crawl directly to the source of a preferred odor. However, insect olfactory navigation is complex. Due to the physiological phenomenon of sensory adaptation, insects often navigate by intermittently entering and exiting odor plumes to reset their olfactory receptors and perceive concentration gradients [32]. Consequently, an insect may enter an arm briefly during random searching or navigation without making a true biological choice. This creates a critical methodological challenge: distinguishing between “accidental intrusion” and “effective choice”. An “effective choice” is defined as a purposeful, directed behavioral response where the insect enters and remains in the odor zone driven by olfactory stimulation, as opposed to a random, transient entry. Currently, criteria for defining a response vary widely, ranging from “the first entry (0 s)” [33,34] to fixed time thresholds such as 15 s [35], 20 s [36], 30 s [15,24], or even 60 s [25,37]. These standards were established based on preliminary experiments or empirical experience, which compromise the comparability and reproducibility of experimental results across the field. To address this methodological issue, this study aims to propose an objective evaluation standard based on the distribution characteristics of the behavioral data itself.

*Trichogramma japonicum* (Ashmead) (Hymenoptera: Trichogrammatidae) is a dominant parasitoid wasp species used for controlling rice stem borers, holding significant value in reducing chemical pesticide use and promoting sustainable field ecology [38,39,40]. The ability of *T. japonicum* to accurately locate hosts via olfactory cues directly determines its parasitism efficiency and control efficiency [41,42,43].

Using *T. japonicum* as a model, we systematically observed its behavioral responses to 20 odorants in a four-arm olfactometer. We found that applying different time criteria to evaluate the preferences of *T. japonicum* yielded different outcomes, highlighting the importance of establishing a unified and objective temporal judgment standard. By analyzing the distribution of its stay duration after first entering an odor field, the 5th percentile of the distribution was set as the statistical threshold for distinguishing accidental intrusion and effective choice. Thus, a continuous stay duration of ≥10s was defined as an effective choice. Applying this newly established criterion, we systematically evaluated the olfactory behavioral response profile of *T. japonicum* to the 20 odorants. Furthermore, oviposition choice experiments were conducted to verify whether the olfactory behavioral preferences determined under this criterion had true biological significance. The results showed consistency between oviposition choice and olfactory behavioral preferences.

## 2. Materials and Methods

### 2.1. Insect Rearing

*Trichogramma japonicum* were mass-reared in glass vials (24 mm in diameter and 95 mm in length) under controlled conditions at 26 ± 1 °C, 70 ± 5% relative humidity, and a 14L:10D photoperiod in the Laboratory of Insect Molecular Ecology and Evolution, Nanjing Agricultural University, with *Corcyra cephalonica* (Stainton) (Lepidoptera: Pyralidae) eggs as the host. Adults were fed with 10% (m/m) sucrose solution before being tested. In all the tests, mated fresh (<12 h old) female wasps were used [44], because parasitoid wasps at this stage exhibit the strongest physiological drive for host-foraging and oviposition, resulting in the most sensitive and robust olfactory responses. The host *C. cephalonica* larvae were reared indoors for multiple generations on a 3:1 mixture of corn flour and soybean flour under conditions of 26 ± 1 °C, 40% relative humidity, and a 14L:10D photoperiod.

### 2.2. Odorants and Preparation

The test odorants (Table 1) were diluted with n-hexane (Shanghai Macklin Biochemical Technology Co., Ltd., Shanghai, China, with a purity of 97%) to prepare compounds at serial concentrations of 0.001, 0.01, 0.1, 1, and 10 μg/μL. Sex pheromone mixtures were also prepared (For *Chilo suppressalis* (Walker) (Lepidoptera: Crambidae): Z11-16:Ald: Z13-18:Ald: Z9-16:Ald = 48: 6: 5 [45,46,47]; for *Cnaphalocrocis medinalis* Guenée (Lepidoptera: Crambidae): Z11-18:Ald: Z13-18:Ald: Z11-18:OH: Z13-18:OH = 3:25:3:3 [48], and for *Ostrinia furnacalis* (Guenée) (Lepidoptera: Crambidae): Z12-14:Ac: E12-14:Ac = 53: 47 [49]).

### 2.3. Odor Preference Experiment

A four-arm olfactometer (18.0 cm × 18.0 cm, and 3.5 cm in height; Nanjing Xuelai Biotechnology Co., Ltd., Nanjing, China; XLM4-120) was used to investigate the behavioral preference of *T. japonicum*. The entire apparatus, connected in sequence using food-grade silicone tubing, consisted of the following components: an air pump → a flow meter → an air purification chamber → odor chamber → the arms of four-arm olfactometer. The olfactometer was equipped with an underlying LED panel (100 W, 6500–7500 K, rated input power: 16 W) as the light source. A total of 10 μL of the odorants or n-hexane was applied to a filter paper strips (3 cm × 2 cm), and the strips were placed into the four odor chambers as follows: two diametrically opposite chambers received the test odorant, while the other two diametrically opposite chambers received n-hexane as controls. The airflow in each arm was set to 100 mL/min [60], because it generates a stable and distinct odor plume without creating excessive turbulence that could disrupt the wasps delicate flight or walking behavior. The olfactometer tests were conducted at 25 ± 1 °C, 70 ± 5% RH. A female *T. japonicum* wasp was released from the central opening of the olfactometer, and its behavior was recorded for 5 min. To prevent odor cross-contamination, after every three wasps were tested, the filter paper strips were removed and discarded. The olfactometer arena and the four odor chambers were wiped thoroughly with 75% ethanol, and then wiped dry with fresh tissue. The system was then purged with clean air at 100 mL/min for 5 min to eliminate residual volatiles. New filter paper strips with freshly applied odorant or n-hexane were used for the subsequent tests. To avoid directional and positional biases, the olfactometer arena was rotated 90° counterclockwise, and the airflow tubing connected to the odor sources was correspondingly repositioned. Thirty wasps were tested per concentration for each of the 20 odorants. A wasp was recorded as having entered an odor zone when its body crossed the dashed line marking the boundary of that zone (Supplementary Figure S1). Number of entries into and the residence time of each entry in each odor zone were analyzed according to a given threshold time. Totally, 3000 *T. japonicum* female wasps were observed.

### 2.4. Olfactory Preference and Effective Residency

During the behavioral observations, we noted *T. japonicum* exhibited oscillatory exploration. Specifically, even towards preferred odors, individuals frequently displayed a repeated exploration pattern—entering the target odor field, briefly leaving, and then returning. Furthermore, some individuals exhibited non-directional movement due to initial environmental stress, indicating that their first choice might not reflect their true preference. Based on these observations, this study posits that only a stay duration that is longer than a given time (threshold time) could be considered as an effective choice. Consequently, relying solely on a single or the first entry as an attraction indicator may not accurately measure their ultimate behavioral tendency [61]. To more objectively quantify their selection preference, this study defined and adopted the attraction rate (AR) as the evaluation metric:

$$\mathrm{AR}\;\left(\%\right)=\frac{\mathrm{Number}\;\mathrm{of}\;\mathrm{effective}\;\mathrm{entries}\;\mathrm{into}\;\mathrm{the}\;\mathrm{test}\;\mathrm{odor}\;\mathrm{field}\;}{\mathrm{Total}\;\mathrm{number}\;\mathrm{of}\;\mathrm{effective}\;\mathrm{entries}\;\mathrm{into}\;\mathrm{all}\;\mathrm{odor}\;\mathrm{fields}\;}\times 100\%$$

To illustrate how the threshold time affects the results of preference behavior, we evaluated the behavior using different thresholds (0, 10, 15, 20, 30, and 60 s).

To establish an objective threshold, we compiled the first stay duration (regardless of the odor zone) of all 3000 female wasps to generate an empirical distribution. The 5th percentile of this distribution was selected as the candidate threshold time based on the convention of rejecting the null hypothesis at a probability level (α) of 5% [62]. A resampling procedure was performed to verify the stability of this threshold by randomly drawing subsamples of various sizes of 30, 50, 80, 100, 200, 300, 400, 500, 1000, 1500, 2000, and 2500 using the RAND () function in Microsoft Excel.

### 2.5. Oviposition Preference

This experiment was conducted in an oviposition selection box [63]. The 2 cm × 2 cm filter papers with 10 μL of host sex pheromone solution were placed on the floor of the odor source chambers at two diagonal corners of the box, while the other two odor source chambers were set with n-hexane (control). Then, after the egg cards were placed in the designated area, five wasps were introduced simultaneously into the oviposition selection box according to a pre-experiment [63]. After 3 h exposure, the wasps were removed and all the egg cards were transferred into plastic cylindrical tubes (one card per tube), and the parasitized eggs were counted three days later according to the color [64]. The oviposition index (OI) ((number of eggs parasitized at treatment − number of eggs parasitized at control)/total number of eggs parasitized) was calculated [65]. The preference of *T. japonicum* between each odorant (0.001, 0.01, 0.1, 1, and 10 μg/μL) and n-hexane were tested, and ten replicates were performed for each of the five concentrations.

### 2.6. Data Analysis

The olfactory choice preferences of *T. japonicum* for different odorants were analyzed using the Chi-square test in GraphPad Prism (version 9.5). The oviposition choice preference was analyzed using a Generalized Liner Mixed Model in IBM SPSS Statistics (version 27) [65]. The OI values were modeled with a Gaussian distribution and an identity link function, as they were continuous and approximately normally distributed (Shapiro–Wilk test, *p* > 0.05). Fixed effects included odorant treatment (test odor vs. n-hexane control), concentration (0.001, 0.01, 0.1, 1, and 10 μg/μL), and their interaction. Box ID was included as a random intercept to account for shared environmental variance within each replicate box, effectively nesting the individual treatment-vs-control comparisons within the replication unit. The model intercept was tested against zero to identify significant preferences (i.e., OI significantly different from 0, indicating preference or repellence). Model fit was assessed by examining Pearson residuals versus fitted values to check for homogeneity of variance, and Q-Q plots of residuals were used to verify approximate normality. The results were visualized using GraphPad Prism (version 9.5).

## 3. Result

### 3.1. Inconsistent Behavioral Results Arising from Different Temporal Judgment Criteria

We systematically evaluated the behavioral preferences of *T. japonicum* towards 20 odorants using different temporal criteria. When 0 s, 15 s, 30 s, and 60 s were used as thresholds for an effective choice, 29% of the tested odorant-concentration combinations (29 out of 100) yielded inconsistent outcomes depending on the threshold applied (Table 2, Table 3 and Table 4). For instance, 10 μg/μL *O. furnacalis* sex pheromone had no attractive effect on *T. japonicum* at thresholds of 0, 15, or 30 s, but was significantly attractive when a 60 s threshold was used (Table 2). Similarly, 10 μg/μL linalool was not repellent at thresholds of 0 or 15 s, but significant repellence was observed at 30 s and 60 s thresholds (Table 4). These results clearly demonstrate that applying different thresholds may directly lead to inconsistent research conclusions.

### 3.2. A Continuous Stay of 10 s or More Constitutes a Valid Olfactory Selection

**Figure 1:**
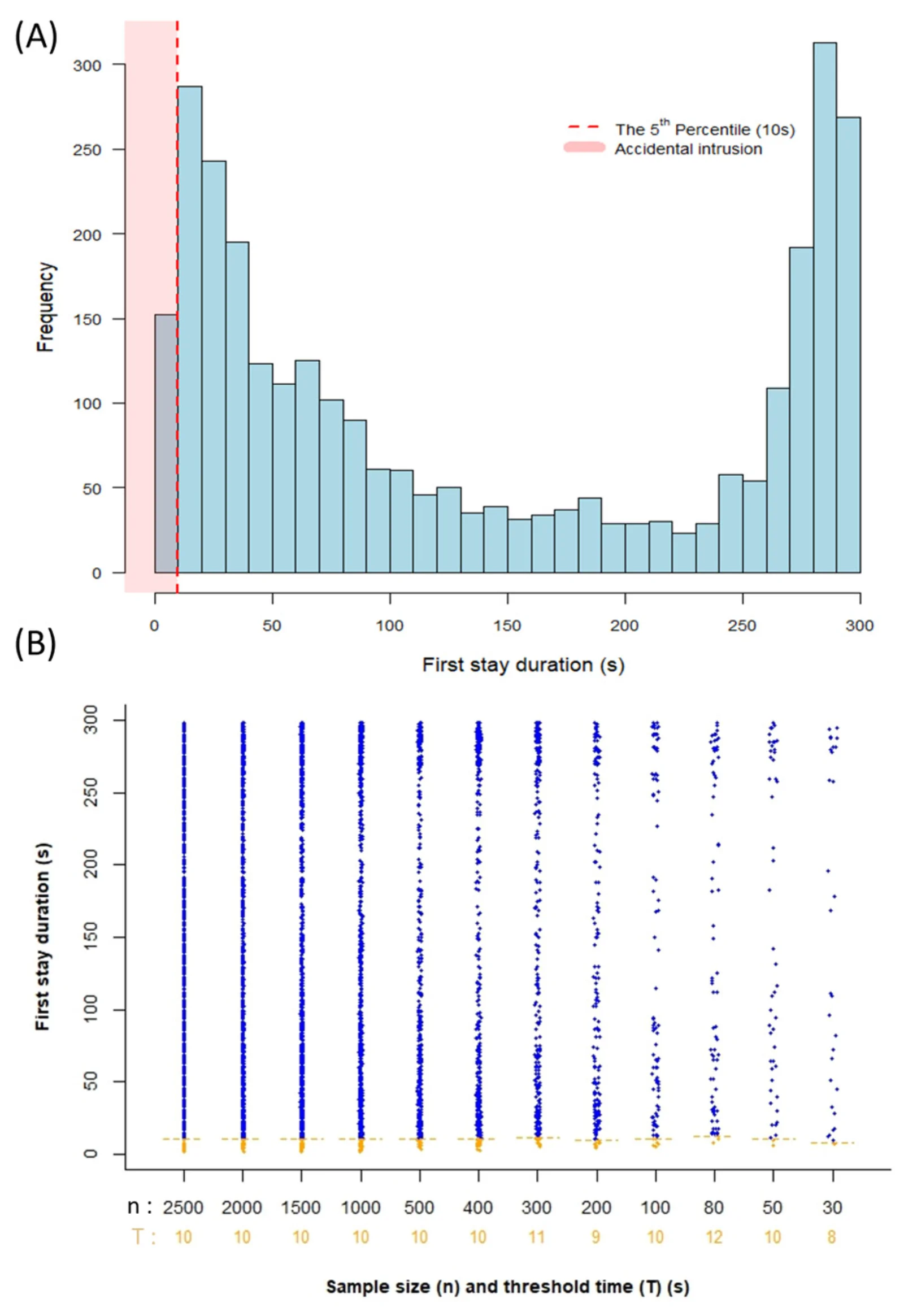
Determination and validation of the behavioral threshold based on the distribution of initial stay duration. (**A**) Frequency distribution of the first stay duration of *T. japonicum* in the first-entered odor zone (n = 3000). Durations < 10 s are classified as accidental intrusion, while those ≥ 10 s are classified as effective choice. (**B**) Threshold time (the 5th percentile) estimated by resampling analysis.

The 5th percentile value for the total sample was 10 s (Figure 1A). The resampling procedure indicated that the 5th percentile remained constant when subsamples were randomly drawn with sample sizes greater than 400, below which the 5th percentile value fluctuated between 8 and 12 s (Figure 1B).

**Figure 2:**
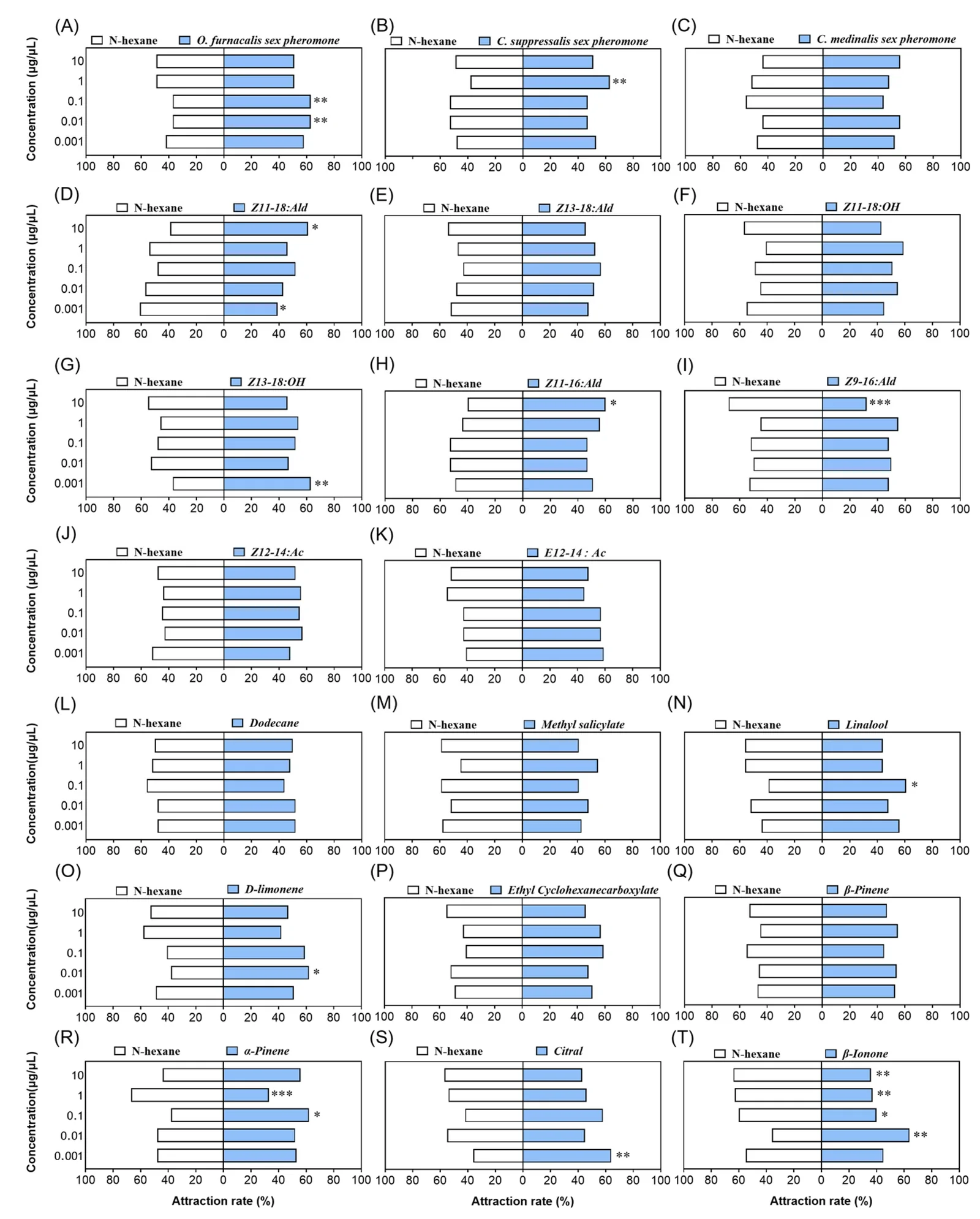
Olfactory behavioral response profile of *T. japonicum* to 20 odorants based on the 10 s threshold. (**A**) *O. furnacalis* sex pheromone; (**B**) *C. suppressalis* sex pheromone; (**C**) *C. medinalis* sex pheromone; (**D**) Z11-18:Ald; (**E**) Z13-18:Ald; (**F**) Z11-18:OH; (**G**) Z13-18:OH; (**H**) Z11-16:Ald; (**I**) Z9-16:Ald; (**J**) Z12-14:Ac; (**K**) E12-14:Ac; (**L**) Dodecane; (**M**) Methyl salicylate; (**N**) Linalool; (**O**) D-limonene; (**P**) Ethyl Cyclohexanecarboxylate; (**Q**) β-Pinene; (**R**) α-Pinene; (**S**) Citral; (**T**) β-Ionone;*: *p* < 0.05, **: *p* < 0.01, ***: *p* < 0.001; Chi-square test.

Based on this, a continuous stay of ≥ 10 s was defined as a valid olfactory selection. Applying the established 10 s criterion, we systematically evaluated the olfactory behavioral responses of *T. japonicum* to 20 different odorants (Figure 2). The results indicated that the sex pheromone of *O. furnacalis* exhibited an attractive effect on *T. japonicum* at concentrations of 0.1 μg/μL or 0.01 μg/μL (Figure 2A). The *C. suppressalis* sex pheromone was attractive at 1 μg/μL (Figure 2B). Specifically, the component Z11-16:Ald (10 μg/μL) attracted *T. japonicum* (Figure 2H), whereas Z9-16:Ald (10 μg/μL) repelled the wasps (Figure 2I). Components of *C. medinalis* sex pheromone, Z11-18:Ald (10 μg/μL) (Figure 2D) and Z13-18:OH (0.001 μg/μL) (Figure 2G) exhibited attractive effects on *T. japonicum*.

Plant-derived volatile compounds triggered distinct behavioral responses of *T. japonicum*, depending on the compound concentration. Notably, both β-ionone (Figure 2T) and α-pinene (Figure 2R) exhibited a typical biphasic dose–response pattern: low concentrations of the two compounds elicited attractive effects, whereas high concentrations elicited avoidance behavior of *T. japonicum*. This phenomenon was particularly pronounced for β-ionone, suggesting its potential role as a significant ecological signal for *T. japonicum* in the environment. Furthermore, citral (0.001 μg/μL) (Figure 2S) and linalool (0.1 μg/μL) (Figure 2N) also attracted *T. japonicum*.

To accurately reflect the potential sample size-dependent variability of the threshold, we performed a subsampling analysis with n = 30, 50 or 80, which yielded a 5th percentile of 8 s, 10 s, and 12 s, respectively (Figure 1B). Subsequently, we re-evaluated the olfactory behavioral data using 8 s and 12 s as candidate thresholds, and the results showed that only 3% of the treatment outcomes were inconsistent with those based on the 10 s threshold (Table 5, Table 6 and Table 7).

### 3.3. Oviposition Choice Behavior Validates Olfactory Preferences

**Figure 3:**
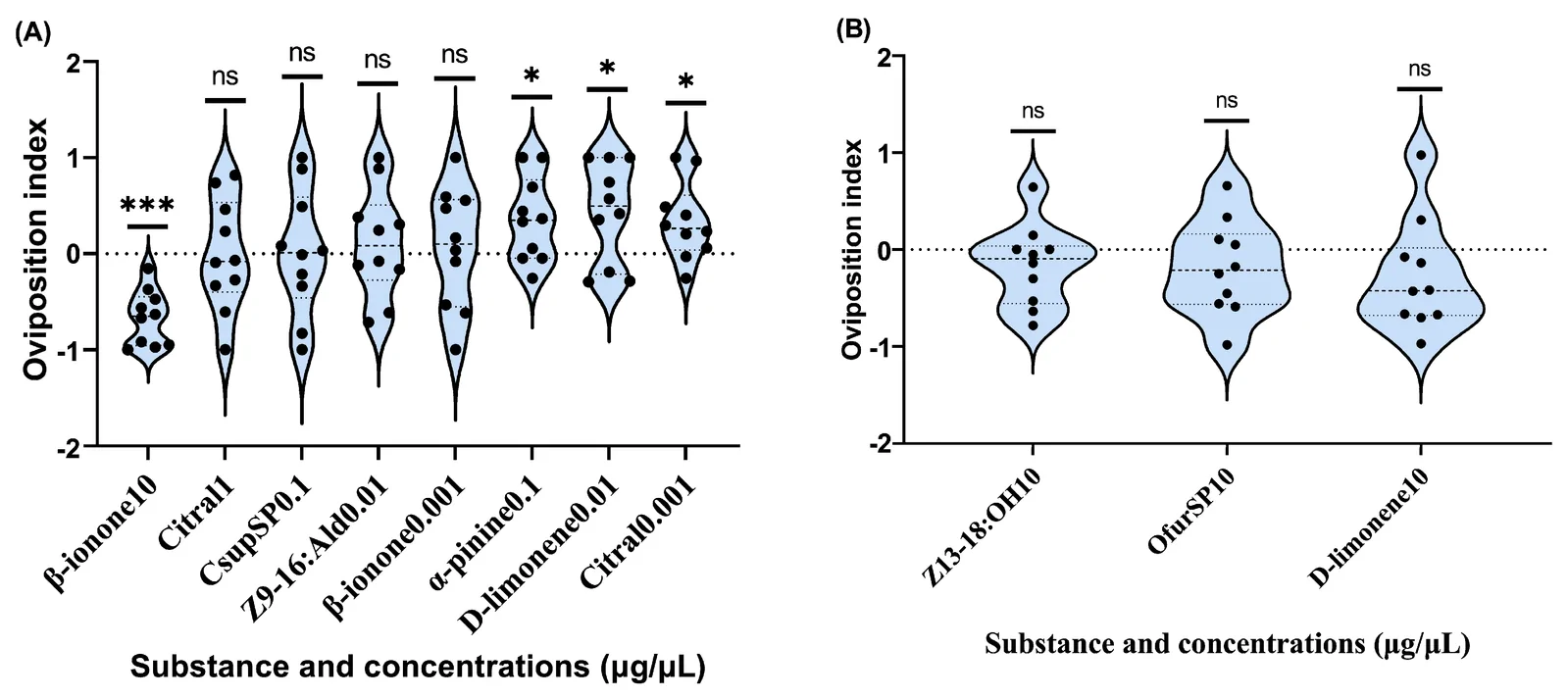
Oviposition choice assay validating olfactory preference. (**A**) Odorants at specific concentrations showing consistent behavioral responses across thresholds (n = 5 × 10). (**B**) Odorants showing inconsistent responses across thresholds (n = 5 × 10). OI > 0 indicates preference, OI < 0 indicates repellence. *: *p* < 0.05, ***: *p* < 0.001, ns: no significant difference (*p* > 0.05, GLMM).

To verify whether the olfactory behavioral preferences have genuine biological significance, we randomly selected seven odorants (eight treatments) at specific concentrations, which had yielded consistent results across different temporal thresholds in the olfactory tests: β-ionone at 10 μg/μL, citral at 1 μg/μL, *C. suppressalis* sex pheromone at 0.1 μg/μL, Z9-16:Ald at 0.001 μg/μL, β-ionone at 0.001 μg/μL, α-pinene at 0.1 μg/μL, D-limonene at 0.01 μg/μL, and citral at 0.01 μg/μL to conduct an oviposition choice assay using the oviposition selection box. The effects of these seven odorants on *T. japonicum* oviposition preference were fully consistent with the results obtained in the four-arm olfactometer (Figure 2 and Figure 3A). Subsequently, we selected three odorants at specific concentrations that had shown inconsistent results when different time thresholds were applied in the four-arm olfactometer: Z13-18:OH at 10 μg/μL, the sex pheromone of *O. furnacalis* at 10 μg/μL, and D-limonene at 10 μg/μL. When the threshold time was set to 10 s, all three odorants had no effect on *T. japonicum* in the olfactometer assay. Correspondingly, these three odorants also showed no effect on *T. japonicum* oviposition preference (Figure 3B). The consistency between oviposition choice and olfactory behavioral preference strongly demonstrates that the olfactory behavior measured using the 10 s criterion can reliably predict the oviposition and host-selection decisions of *T. japonicum* under conditions more closely resembling natural scenarios.

## 4. Discussion

This study systematically investigated the impact of behavioral evaluation criteria on insect preference results. Using *T. japonicum* as a model, we successfully established a data-driven temporal standard. Data analysis revealed that when different thresholds ranging from 0 s to 60 s were applied, 29% of the test results varied with the change in the threshold time. This inconsistency, which is beyond random error, underscores the lack of objectivity in current subjective time standards. To address this issue, we applied statistical methods to the distribution of extensive behavioral data, identifying an objective temporal threshold that effectively distinguished accidental intrusion from effective choice, and established a data-driven criterion for olfactory behavior studies.

In experimental observations, when an insect first enters a specific odor field, it was considered that this field exerts an attractive effect on the insect. However, a brief, accidental intrusion into an odor field should not be considered as a valid choice. Thus, a genuine choice is defined by a stay duration exceeding that of a mere accidental intrusion, and a temporal threshold needed to be identified to distinguish accidental intrusion from effective choice. We systematically observed and recorded the behavior of 3000 *T. japonicum* wasps in a four-arm olfactometer, focusing on the timing of their entry into and exit from each odor zone. Within the empirical distribution of the first stay duration, we posited that behaviors with extremely short durations represent accidental intrusions resulting from random movement. To quantify this level of occasionality, we employed the statistical concept of percentiles [66]. Specifically, the 5th percentile was selected as the threshold [67,68].

The 5th percentile value is relatively stable and independent of sample size when a sufficient number of individuals are investigated. By analyzing the empirical distribution of the first stay duration for 3000 *T. japonicum* individuals and defining the behavioral threshold using the 5th percentile, our method treats the shortest 5% of durations as accidental intrusions. Consequently, an intentional choice is objectively defined as a continuous stay duration of ≥10 s. We validated this temporal standard through resampling, which confirmed the stability of the 10 s value across different subsample sizes with sample sizes greater than 400. This indicates that 10 s is a robust statistical characteristic independent of any specific subsample, although the 5th percentile was artificially selected. Crucially, further validation through oviposition choice experiments demonstrated that olfactory behavioral data obtained using the 10 s standard reliably predicted the oviposition decisions of *T. japonicum* wasps in near-natural conditions. This strongly confirms the ecological relevance and predictive value of the behavioral criterion established in this study, thereby providing a scientific basis for utilizing behavioral manipulation to enhance the efficacy of *Trichogramma* in biological control.

Derived from the intrinsic distribution of the experimental data and validated for reliability, this standard significantly enhances the objectivity and scientific rigor of behavioral assessment, and the 3% inconsistence is acceptable even when the sample is as small as 30 replications. However, it must be emphasized that when applying such data-driven methods to determine behavioral thresholds, sample size must be carefully evaluated [69] and reported to avoid distortion of threshold estimates due to insufficient sampling, which would compromise the reliability of research conclusions [70].

The behavioral responses of *Trichogramma* are complex and are modulated not only by odorant concentration but also by its chemical composition. In the olfactory preference study based on the 10 s threshold, we found that while *O. furnacalis* sex pheromone attracted *T. japonicum* (Figure 2A), its individual components, both Z12-14:Ac (Figure 2J) and E12-14:Ac (Figure 2K), did not. This suggests that the attraction relies on the combined action of both compounds, underscoring the precision of chemical communication. Z11-16:Ald (Figure 2H) attracted *T. japonicum*, whereas Z9-16:Ald (Figure 2I) repelled it. However, the *C. suppressalis* sex pheromone exhibited an attractive effect (Figure 2B). We propose that this is because the attractive effect of the major component, Z11-16:Ald (Figure 2H), dominates the repellent effect of the minor component, Z9-16:Ald (Figure 2I). Among the *C. medinalis* sex pheromone components, 10 μg/μL Z11-18:Ald (Figure 2D) and 0.001 μg/μL Z13-18:OH (Figure 2G) attracted *T. japonicum*. As these components are minor constituents in the full pheromone blend, the complete *C. medinalis* sex pheromone mixture did not show attraction to *T. japonicum*. Regarding plant-derived volatile compounds, both β-ionone (Figure 2T) and α-pinene (Figure 2R) exhibited a biphasic dose–response: attraction at low concentrations and repellence at high concentrations. This bidirectional response reflects the precision and complexity of plant chemical communication [65,71,72]. Low concentrations of plant volatiles may signal healthy or mildly stressed plants, often induced by pest oviposition [73], indicating to *T. japonicum* the potential presence of hosts in the vicinity, making the area worth exploring [74]. Conversely, high concentrations of herbivore-induced plant volatiles likely indicated severe plant stress, often due to larval feeding after egg hatching, leading to the release of large quantities of secondary metabolites [75]. At this stage, the pests have entered the larval stage, which is unsuitable for oviposition of the egg parasitoid *Trichogramma,* thus explaining the repellent effect—a key distinction between egg parasitoids and larval or pupal parasitoids [71,76,77]. Furthermore, specific concentrations of citral, D-limonene, and linalool also attracted *T. japonicum*, collectively forming a potential attractant portfolio alongside β-ionone and α-pinene.

The 17 compounds listed in Table 1 are all derived from ecological contexts relevant to *Trichogramma* habitats. Among them, some have been previously investigated in *T. japonicum* or other *Trichogramma* species, while the remaining compounds are reported for the first time in this study. For example, Tian et al. identified D-limonene and α-pinene as the dominant oviposition-induced plant volatiles emitted by rice plants infested with *C. medinalis* eggs, and demonstrated that synthetic versions of these two monoterpenes were highly attractive to *T. japonicum* females [41]. In addition, Bai et al. [78] reported that the sex pheromone of *C. suppressalis* attracted *T. japonicum* females in Y-tube olfactometer assays. Our results are largely consistent with these findings, as we also observed a clear olfactory preference of *T. japonicum* for the sex pheromone of *C. suppressalis*. However, for most of the compounds listed in Table 1—particularly the individual sex pheromone components (e.g., Z11-16:Ald, Z13-18:Ald, Z9-16:Ald, etc.) and certain plant volatiles (e.g., β-ionone, citral, ethyl cyclohexanecarboxylate, and n-dodecane)—although some have been sporadically reported in other *Trichogramma* species, direct evidence regarding their olfactory effects on *T. japonicum* remains scarce. Therefore, this study provides novel empirical data on the behavioral responses of *T. japonicum* to these compounds, contributing to a more comprehensive understanding of the chemical ecology of this important biological control agent.

While the 60 s threshold yields more “statistically significant” results, we argue this does not equate to higher true sensitivity for olfactory choice. A 60 s threshold effectively forces a prolonged observation, which measures arrestment (the tendency to stay) rather than the initial attraction/choice. Furthermore, requiring a 60 s stay artificially filters out many valid, quick choices, thereby skewing the denominator in AR calculations and potentially generating false positives. The 10 s threshold, derived from empirical data distribution, accurately captures the true “decision-making” moment without confounding attraction with long-term arrestment.

It is important to emphasize that the 10 s threshold specified in this study is an optimized standard for the specific context of *T. japonicum* in a four-arm olfactometer and should not be directly generalized as a universal constant across different experiments or species. The behavioral threshold may vary depending on species-specific locomotor activity, body size, olfactory sensitivity, and experimental parameters such as airflow rate, arena dimensions, and odorant concentration. Nevertheless, the true universal contribution of this study is not the specific 10 s value per se, but rather the methodological paradigm it demonstrates: shifting from subjective, empirically assigned thresholds to a data-driven approach that defines effective behavior based on the statistical distribution of behavioral data, using the 5th percentile to distinguish purposeful choices from accidental intrusions. This conceptual and statistical framework is broadly applicable to olfactory behavior studies across diverse insect taxa and can be adapted to various experimental systems, including Y-tube olfactometers, two-choice arenas, and wind tunnels, provided that sufficient behavioral data are collected to establish a reliable empirical distribution of the relevant behavioral parameter. Therefore, we recommend that future studies reporting insect behavioral preferences should not only present results but also explicitly detail the criteria used to define effective behavior and their statistical or biological rationale.

In summary, this study proposed and validated an objective behavioral assessment criterion grounded in data-driven framework and successfully implemented to investigate the olfactory preference patterns in *T. japonicum.* Our findings not only elucidate the olfactory behavioral responses of *T. japonicum* to specific odorants, laying a robust scientific basis for refining mass-rearing protocols of and field biocontrol tactics with the wasp, but also provide methodological underpinnings for constructing a repeatable paradigm for olfactory behavioral assays across other beneficial predatory and parasitic insects. This advancement further improves the reliability and cross-study comparability of relevant chemical ecology research outputs.

## Figures and Tables

**Table 1:** List of odorant compounds tested for olfactory behavioral assays in *Trichogramma japonicum*.

Compound	Producer (Purity%)	CAS	Storage Condition	Odor Source	Reference
α-pinene	Shanghai Macklin Biochemical Technology Co., Ltd., Shanghai, China (98)	80-56-8	Room temperature	Rice volatile	[41,50]
β-ionone	Shanghai Jizhi Biochemical Technology Co., Ltd., Shanghai, China (97)	14901-07-6	4 °C	Aromatic volatile	[51,52]
β-pinene	Anpel Laboratory Technologies (Shanghai) Inc., Shanghai, China (94)	18172-67-3	4 °C	Masson volatile	[50]
Citral	Beijing MREDA Technology Co., Ltd., Beijing, China (95)	5392-40-5	Room temperature	Aromatic volatile	[53]
D-limonene	Shanghai Macklin Biochemical Technology Co., Ltd., Shanghai, China (95)	7705-14-8	4 °C	Rice volatile	[41]
Ethyl Cyclohexanecarboxylate	Beijing Kaiguo Technology Co., Ltd., Beijing China (98)	3289-28-9	Room temperature	Rice volatile	[54]
Linalool	Shanghai Macklin Biochemical Technology Co., Ltd., Shanghai, China (95)	78-70-6	Room temperature	Rice volatile	[55]
Methyl salicylate	Beijing Kaiguo Technology Co., Ltd., Beijing, China (95)	119-36-8	Room temperature	Rice volatile	[55,56,57,58,59]
N-dodecane	Shanghai Dibo Biotechnology Co., Ltd., Shanghai, China (≥99)	112-40-3	Room temperature	Rice volatile	[59]
E12-14: Ac	Jiangsu Ninglu Technology Co., Ltd., Nanjing, China (95)	35153-21-0	−20 °C	Sex pheromone component	[49]
Z9-16: Ald	Jiangsu Ninglu Technology Co., Ltd., Nanjing, China (95)	56219-04-6	−20 °C		[46,47]
Z11-16: Ald	Jiangsu Ninglu Technology Co., Ltd., Nanjing, China (95)	10,064-53-8	−20 °C		[49]
Z11-18: Ald	Jiangsu Ninglu Technology Co., Ltd., Nanjing, China (95)	53939-28-9	−20 °C		[48]
Z11-18: OH	Jiangsu Ninglu Technology Co., Ltd., Nanjing, China (95)	62972-93-4	−20 °C		[48]
Z12-14:Ac	Jiangsu Ninglu Technology Co., Ltd., Nanjing, China (95)	35153-20-9	−20 °C		[49]
Z13-18: Ald	Jiangsu Ninglu Technology Co., Ltd., Nanjing, China (95)	58594-45-9	−20 °C		[45,48]
Z13-18: OH	Jiangsu Ninglu Technology Co., Ltd., Nanjing, China (95)	69820-27-5	−20 °C		[48]

**Table 2:** The attraction rates of host sex pheromone to *T. japonicum* under different threshold times (n = 30).

Host	Threshold Time (s)	Concentration (μg/μL)									
		10		1		0.1		0.01		0.001	
		AR † (%)	*p*	AR (%)	*p*	AR (%)	*p*	AR (%)	*p*	AR (%)	*p*
*Ostrinia furnacalis*	0	47	0.5485	51	0.8415	63	0.0093	63	0.0093	57	0.1615
	15	50	>0.9999	49	0.8415	64	0.0051	62	0.0164	57	0.1615
	20	50	>0.9999	48	0.6892	63	0.0093	64	0.0051	50	>0.9999
	30	49	0.8415	50	>0.9999	66	0.0014	64	0.0051	58	0.1096
	60	70	<0.0001	58	0.1096	70	<0.0001	72	<0.0001	58	0.1096
*Chilo suppressalis*	0	51	0.8415	64	0.0051	49	0.8415	48	0.6892	53	0.5485
	15	53	0.5485	63	0.0093	49	0.8415	47	0.5485	53	0.5485
	20	57	0.1615	61	0.0278	49	0.8415	51	0.8415	53	0.5485
	30	54	0.4237	68	0.0003	48	0.6892	48	0.6892	53	0.5485
	60	58	0.1096	59	0.0719	44	0.2301	51	0.8415	59	0.0719
*Cnaphalocrocis medinalis*	0	58	0.1096	46	0.4237	43	0.1615	55	0.3173	51	0.8415
	15	59	0.0719	46	0.4237	42	0.1096	56	0.2301	51	0.8415
	20	58	0.1096	46	0.4237	44	0.2301	56	0.2301	51	0.8415
	30	59	0.0719	45	0.3173	47	0.5485	54	0.4237	47	0.5485
	60	64	0.0051	37	0.0093	52	0.6892	57	0.1615	45	0.3173

† AR: attraction rate. *Trichogramma* significantly preferred (AR > 50%) or avoided (AR < 50%) the odorant if *p* < 0.05 (Chi-square test).

**Table 3:** The attraction rates of sex pheromone components to *T. japonicum* under different threshold times (n = 30).

Odorant	Threshold Time (s)	Concentration μg/μL									
		10		1		0.1		0.01		0.001	
		AR † (%)	*p*	AR (%)	*p*	AR (%)	*p*	AR (%)	*p*	AR (%)	*p*
Z11-16:Ald	0	50	>0.9999	53	0.5485	48	0.6892	47	0.5485	52	0.6892
	15	51	0.8415	53	0.5485	51	0.8415	46	0.4237	58	0.1096
	20	51	0.8415	55	0.3173	46	0.4237	48	0.6892	58	0.1096
	30	53	0.5485	49	0.8415	43	0.1615	50	>0.9999	59	0.0719
	60	53	0.5485	52	0.6892	42	0.1096	52	0.6892	60	0.0455
Z13-18:Ald	0	46	0.4237	54	0.4237	59	0.0719	53	0.5485	48	0.6892
	15	45	0.3173	54	0.4237	61	0.0278	53	0.5485	51	0.8415
	20	48	0.6892	55	0.3173	58	0.1096	50	>0.9999	51	0.8415
	30	43	0.1615	54	0.4237	60	0.0455	50	>0.9999	52	0.6892
	60	42	0.1096	63	0.0093	65	0.0027	48	0.6892	58	0.1096
Z9-16:Ald	0	32	0.0003	55	0.3173	46	0.4237	50	>0.9999	51	0.8415
	15	31	0.0001	56	0.2301	46	0.4237	45	0.3173	50	>0.9999
	20	29	<0.0001	57	0.1615	46	0.4237	45	0.3173	53	0.5485
	30	27	<0.0001	56	0.2301	43	0.1615	48	0.6892	49	0.8415
	60	27	<0.0001	52	0.6892	45	0.3173	48	0.6892	53	0.5485
Z11-18:Ald	0	61	0.0278	48	0.6892	53	0.5485	44	0.2301	40	0.0455
	15	61	0.0278	49	0.8415	53	0.5485	47	0.5485	38	0.0164
	20	59	0.0719	49	0.8415	51	0.8415	44	0.2301	34	0.0014
	30	57	0.1615	50	>0.9999	42	0.1096	46	0.4237	39	0.0278
	60	53	0.5485	47	0.5485	47	0.5485	46	0.4237	39	0.0278
Z11-18:OH	0	41	0.0719	57	0.1615	51	0.8415	48	0.6892	44	0.2301
	15	43	0.0001	59	0.0719	51	0.8415	47	0.5485	45	0.3173
	20	42	0.1096	58	0.1096	51	0.8415	50	>0.9999	45	0.3173
	30	41	0.0719	62	0.0164	52	0.6892	48	0.6892	47	0.5485
	60	34	0.0014	63	0.0093	51	0.8415	53	0.5485	46	0.4237
Z13-18:OH	0	42	0.1096	51	0.8415	48	0.6892	42	0.1096	63	0.0093
	15	42	0.1096	51	0.8415	49	0.8415	44	0.2301	62	0.0164
	20	42	0.1096	52	0.6892	47	0.5485	42	0.1096	65	0.0027
	30	38	0.0164	51	0.8415	48	0.6892	39	0.0278	66	0.0014
	60	33	0.0007	50	>0.9999	49	0.8415	40	0.0455	65	0.0027
Z12-14Ac	0	51	0.8415	54	0.4237	55	0.3173	56	0.2301	48	0.6892
	15	51	0.8415	60	0.0455	53	0.5485	54	0.4237	49	0.8415
	20	52	0.6892	61	0.0278	54	0.4237	52	0.6892	50	>0.9999
	30	53	0.5485	57	0.1615	56	0.2301	49	0.8415	50	>0.9999
	60	53	0.5485	59	0.0719	47	0.5485	50	>0.9999	50	>0.9999
E12-14Ac	0	45	0.3173	45	0.3173	55	0.3173	55	0.3173	59	0.0719
	15	48	0.6892	44	0.2301	58	0.1096	61	0.0278	58	0.1096
	20	48	0.6892	46	0.4237	60	0.0455	60	0.0455	58	0.1096
	30	51	0.8415	44	0.2301	60	0.0455	61	0.0278	59	0.0719
	60	52	0.6892	43	0.1615	57	0.1615	62	0.0164	63	0.0093

† AR: attraction rate. *Trichogramma* significantly preferred (AR > 50%) or avoided (AR < 50%) the odorant if *p* < 0.05 (Chi-square test).

**Table 4:** The attraction rates of plant-derived volatile compounds to *T. japonicum* under different threshold times (n = 30).

Odorant	Threshold Time (s)	Concentration μg/μL									
		10		1		0.1		0.01		0.001	
		AR † (%)	*p*	AR (%)	*p*	AR (%)	*p*	AR (%)	*p*	AR (%)	*p*
α-pinene	0	58	0.1096	36	0.0051	62	0.0164	53	0.5485	51	0.8415
	15	56	0.2301	34	0.0014	60	0.0455	48	0.6892	54	0.4237
	20	55	0.3173	33	0.0007	61	0.0278	48	0.6892	53	0.5485
	30	58	0.1096	36	0.0051	63	0.0093	47	0.5485	49	0.8415
	60	56	0.2301	34	0.0014	61	0.0278	50	>0.9999	46	0.4237
β-ionone	0	36	0.0051	36	0.0051	40	0.0455	56	0.2301	46	0.4237
	15	32	0.0003	36	0.0051	35	0.0027	57	0.1615	48	0.6892
	20	33	0.0007	35	0.0027	38	0.0164	55	0.3173	48	0.6892
	30	30	<0.0001	30	<0.0001	37	0.0093	58	0.1096	45	0.3173
	60	27	<0.0001	27	<0.0001	42	0.1096	54	0.4237	45	0.3173
β-pinene	0	45	0.3173	53	0.5485	43	0.1615	48	0.6892	51	0.8415
	15	48	0.6892	40	0.0455	49	0.8415	44	0.2301	46	0.4237
	20	46	0.4237	54	0.4237	44	0.2301	45	0.3173	47	0.5485
	30	44	0.2301	46	0.4237	43	0.1615	46	0.4237	52	0.6892
	60	45	0.3173	47	0.5485	42	0.1096	44	0.2301	55	0.3173
Citral	0	44	0.2301	48	0.6892	57	0.1615	45	0.3173	64	0.0051
	15	48	0.6892	46	0.4237	56	0.2301	43	0.1615	64	0.0051
	20	43	0.1615	48	0.6892	55	0.3173	47	0.5485	64	0.0051
	30	45	0.3173	48	0.6892	59	0.0719	41	0.0719	64	0.0051
	60	36	0.0051	48	0.6892	54	0.4237	38	0.0164	60	0.0455
D-limonene	0	48	0.6892	39	0.0278	58	0.1096	64	0.0051	53	0.5485
	15	49	0.8415	42	0.1096	57	0.1615	62	0.0164	48	0.6892
	20	55	0.3173	41	0.0719	59	0.0719	62	0.0164	50	>0.9999
	30	47	0.5485	43	0.1615	59	0.0719	60	0.0455	49	0.8415
	60	39	0.0278	45	0.3173	64	0.0051	61	0.0278	45	0.3173
N-dodecane	0	47	0.5485	48	0.6892	44	0.2301	52	0.6892	53	0.5485
	15	48	0.6892	49	0.8415	43	0.1615	47	0.5485	52	0.6892
	20	46	0.4237	52	0.6892	44	0.2301	48	0.6892	52	0.6892
	30	48	0.6892	48	0.6892	43	0.1615	54	0.4237	49	0.8415
	60	57	0.1615	46	0.4237	42	0.1096	50	>0.9999	53	0.5485
Ethyl Cyclohexanecarboxylate	0	50	>0.9999	58	0.1096	59	0.0719	49	0.8415	50	>0.9999
	15	57	0.1615	58	0.1096	58	0.1096	48	0.6892	51	0.8415
	20	47	0.5485	61	0.0278	59	0.0719	49	0.8415	51	0.8415
	30	49	0.8415	59	0.0719	61	0.0278	50	>0.9999	49	0.8415
	60	46	0.4237	61	0.0278	62	0.0164	53	0.5485	53	0.5485
Linalool	0	44	0.2301	45	0.3173	60	0.0455	48	0.6892	56	0.2301
	15	42	0.1096	43	0.1615	60	0.0455	44	0.2301	58	0.1096
	20	42	0.1096	43	0.1615	59	0.0719	46	0.4237	55	0.3173
	30	38	0.0164	44	0.2301	61	0.0278	43	0.1615	51	0.8415
	60	36	0.0051	41	0.0719	62	0.0164	35	0.0027	50	>0.9999
Methyl salicylate	0	41	0.0719	54	0.4237	39	0.0278	47	0.5485	44	0.2301
	15	40	0.0455	55	0.3173	38	0.0164	48	0.6892	41	0.0719
	20	40	0.0455	55	0.3173	38	0.0164	51	0.8415	43	0.1615
	30	38	0.0164	57	0.1615	38	0.0164	51	0.8415	47	0.5485
	60	32	0.0003	50	>0.9999	35	0.0027	47	0.5485	46	0.4237

† AR: attraction rate. *Trichogramma* significantly preferred (AR > 50%) or avoided (AR < 50%) the odorant if *p* < 0.05 (Chi-square test).

**Table 5:** The attraction rates of host sex pheromone to *T. japonicum* under 8, 10 or 12-sthreshold time (n = 30).

Host	Threshold Time (s)	Concentration (μg/μL)									
		10		1		0.1		0.01		0.001	
		AR † (%)	*p*	AR (%)	*p*	AR (%)	*p*	AR (%)	*p*	AR (%)	*p*
*Ostrinia furnacalis*	8	46	0.4237	51	0.8415	63	0.0093	63	0.0093	48	0.6892
	10	47	0.5485	50	>0.9999	63	0.0093	63	0.0093	48	0.6892
	12	46	0.4237	48	0.6892	64	0.0051	63	0.0093	49	0.8415
*Chilo suppressalis*	8	53	0.5485	63	0.0093	49	0.8415	50	>0.9999	53	0.5485
	10	53	0.5485	63	0.0093	50	>0.9999	50	>0.9999	52	0.6892
	12	52	0.6892	62	0.0164	50	>0.9999	50	>0.9999	52	0.6892
*Cnaphalocrocis medinalis*	8	59	0.0719	46	0.4237	43	0.1615	55	0.3173	53	0.5485
	10	59	0.0719	46	0.4237	43	0.1615	54	0.4237	53	0.5485
	12	58	0.1096	46	0.4237	43	0.1615	54	0.4237	53	0.5485

† AR: attraction rate. *Trichogramma* significantly preferred (AR > 50%) or avoided (AR < 50%) the odorant if *p* < 0.05 (Chi-square test).

**Table 6:** The attraction rates of sex pheromone components to *T. japonicum* under 8, 10 or 12 s threshold time (n = 30).

Odorant	Threshold Time (s)	Concentration (μg/μL)									
		10		1		0.1		0.01		0.001	
		AR † (%)	*p*	AR (%)	*p*	AR (%)	*p*	AR (%)	*p*	AR (%)	*p*
Z11-16:Ald	8	48	0.6892	55	0.3173	48	0.6892	47	0.5485	53	0.5485
	10	49	0.8415	53	0.5485	48	0.6892	47	0.5485	53	0.5485
	12	52	0.6892	53	0.5485	49	0.8415	47	0.5485	56	0.2301
Z13-18:Ald	8	48	0.6892	54	0.4237	58	0.1096	54	0.4237	48	0.6892
	10	48	0.6892	54	0.4237	58	0.1096	52	0.6892	48	0.6892
	12	49	0.8415	53	0.5485	58	0.1096	52	0.6892	49	0.8415
Z9-16:Ald	8	32	0.0003	56	0.2301	45	0.3173	48	0.6892	53	0.5485
	10	32	0.0003	56	0.2301	46	0.4237	46	0.4237	52	0.6892
	12	32	0.0003	56	0.2301	46	0.4237	45	0.3173	53	0.5485
Z11-18:Ald	8	61	0.0278	48	0.6892	52	0.6892	44	0.2301	35	0.0027
	10	61	0.0278	48	0.6892	52	0.6892	44	0.2301	35	0.0027
	12	61	0.0278	48	0.6892	52	0.6892	43	0.1615	35	0.0027
Z11-18:OH	8	41	0.0719	57	0.1615	51	0.8415	50	>0.9999	44	0.2301
	10	44	0.2301	59	0.0719	51	0.8415	50	>0.9999	45	0.3173
	12	42	0.1096	58	0.1096	51	0.8415	50	>0.9999	45	0.3173
Z13-18:OH	8	42	0.1096	54	0.4237	48	0.6892	40	0.0455	63	0.0093
	10	42	0.1096	54	0.4237	48	0.6892	40	0.0455	63	0.0093
	12	42	0.1096	51	0.8415	48	0.6892	41	0.0719	63	0.0093
Z12-14Ac	8	51	0.8415	54	0.4237	55	0.3173	56	0.2301	48	0.6892
	10	51	0.8415	56	0.2301	54	0.4237	56	0.2301	49	0.8415
	12	51	0.8415	59	0.0719	52	0.6892	56	0.2301	49	0.8415
E12-14Ac	8	46	0.4237	45	0.3173	54	0.4237	55	0.3173	59	0.0719
	10	46	0.4237	45	0.3173	55	0.3173	55	0.3173	59	0.0719
	12	48	0.6892	46	0.4237	58	0.1096	57	0.5485	60	0.0455

† AR: attraction rate. *Trichogramma* significantly preferred (AR > 50%) or avoided (AR < 50%) the odorant if *p* < 0.05 (Chi-square test).

**Table 7:** The attraction rates of plant-derived volatile compounds to *T. japonicum* under 8, 10 or 12 s threshold time (n = 30).

Compound	Threshold Time (s)	Concentration (μg/μL)									
		10		1		0.1		0.01		0.001	
		AR † (%)	*p*	AR (%)	*p*	AR (%)	*p*	AR (%)	*p*	AR (%)	*p*
α-pinene	8	55	0.3173	33	0.0007	62	0.0164	52	0.6892	53	0.5485
	10	53	0.5485	33	0.0007	61	0.0278	52	0.6892	53	0.5485
	12	53	0.5485	33	0.0007	61	0.0278	50	>0.9999	52	0.6892
β-ionone	8	36	0.0051	36	0.0051	40	0.0455	56	0.2301	46	0.4237
	10	35	0.0027	36	0.0051	40	0.0455	58	0.1096	46	0.4237
	12	33	0.0007	36	0.0051	40	0.0455	58	0.1096	46	0.4237
β-pinene	8	45	0.3173	58	0.1096	43	0.1615	47	0.5485	47	0.5485
	10	45	0.3173	58	0.1096	43	0.1615	47	0.5485	47	0.5485
	12	44	0.2301	58	0.1096	45	0.3173	45	0.3173	47	0.5485
Citral	8	44	0.2301	46	0.4237	55	0.3173	46	0.4237	64	0.0051
	10	44	0.2301	46	0.4237	55	0.3173	46	0.4237	64	0.0051
	12	44	0.2301	46	0.4237	55	0.3173	42	0.1096	64	0.0051
D-limonene	8	51	0.8415	40	0.0455	61	0.0278	64	0.0051	51	0.8415
	10	53	0.5485	42	0.1096	61	0.0278	64	0.0051	51	0.8415
	12	54	0.4237	42	0.1096	61	0.0278	62	0.0164	50	>0.9999
N-dodecane	8	46	0.4237	51	0.8415	44	0.2301	48	0.6892	53	0.5485
	10	48	0.6892	53	0.5485	44	0.2301	48	0.6892	52	0.6892
	12	48	0.6892	53	0.5485	43	0.1615	48	0.6892	52	0.6892
Ethyl Cyclohexanecarboxylate	8	46	0.4237	57	0.1615	58	0.1096	48	0.6892	51	0.8415
	10	46	0.4237	57	0.1615	58	0.1096	48	0.6892	51	0.8415
	12	45	0.3173	58	0.1096	58	0.1096	48	0.6892	51	0.8415
Linalool	8	41	0.0719	44	0.2301	60	0.0455	47	0.5485	57	0.1615
	10	39	0.0278	44	0.2301	60	0.0455	46	0.4237	57	0.1615
	12	40	0.0455	44	0.2301	60	0.0455	44	0.2301	59	0.0719
Methyl salicylate	8	40	0.0455	54	0.4237	41	0.0719	47	0.5485	40	0.0455
	10	40	0.0455	55	0.3173	39	0.0278	48	0.6892	40	0.0455
	12	40	0.0455	55	0.3173	39	0.0278	48	0.6892	41	0.0719

† AR: attraction rate. *Trichogramma* significantly preferred (AR > 50%) or avoided (AR < 50%) the odorant if *p* < 0.05 (Chi-square test).

## Data Availability

The original contributions presented in this study are included in the article/Supplementary Material. Further inquiries can be directed to the corresponding author.

## Supplementary Materials

The following supporting information can be downloaded at: https://www.mdpi.com/article/10.3390/insects17080798/s1, Figure S1: Schematic diagram of the four-arm olfactometer setup.
